# Hospice use and one-year survivorship of residents in long-term care facilities in Canada: a cohort study

**DOI:** 10.1186/s12904-019-0480-z

**Published:** 2019-11-12

**Authors:** Beibei Xiong, Shannon Freeman, Davina Banner, Lina Spirgiene

**Affiliations:** 1 School of Health Sciences, University of Northern British Columbia, Prince George, British Columbia Canada; 2 School of Nursing, University of Northern British Columbia, 333 University Way, Prince George, British Columbia V2N 4Z9 Canada; 3 Northern Medical Program, University of Northern British Columbia, Prince George, British Columbia Canada; 4 Department of Nursing and Care, Medical Academy, Lithuanian University of Health Sciences, Kaunas, Lithuania

**Keywords:** Hospice care, End-of life care, Long-term care facility, Nursing home, interRAI minimum data set

## Abstract

**Background:**

Hospice care is designed for persons in the final phase of a terminal illness. However, hospice care is not used appropriately. Some persons who do not meet the hospice eligibility receive hospice care, while many persons who may have benefitted from hospice care do not receive it. This study aimed to examine the characteristics of, and one-year survivorship among, residents who received hospice care versus those who did not in long-term care facilities (LTCFs) in Canada.

**Methods:**

This retrospective cohort study used linked health administrative data from the Canadian Continuing Reporting System (CCRS) and the Discharge Abstract Database (DAD). All persons who resided in a LTCF and who had a Resident Assessment Instrument Minimum Data Set Version 2.0 (RAI-MDS 2.0) assessment in the CCRS database between Jan. 1st, 2015 and Dec 31st, 2015 were included in this study (*N* = 185,715). Death records were linked up to Dec 31th, 2016. Univariate, bivariate and multivariate analyses were performed.

**Results:**

The reported hospice care rate in LTCFs is critically low (less than 3%), despite one in five residents dying within 3 months of the assessment. Residents who received hospice care and died within 1 year were found to have more severe and complex health conditions than other residents. Compared to those who did not receive hospice care but died within 1 year, residents who received hospice care and were alive 1 year following the assessment were younger (a mean age of 79.4 [+ 13.5] years vs. 86.5 [+ 9.2] years), more likely to live in an urban LTCF (93.2% vs. 82.6%), had a higher percentage of having a diagnosis of cancer (50.7% vs. 12.9%), had a lower percentage of having a diagnosis of dementia (30.2% vs. 54.5%), and exhibited more severe acute clinical conditions.

**Conclusions:**

The actual use of hospice care among LTCF residents is very poor in Canada. Several factors emerged as potential barriers to hospice use in the LTCF population including ageism, rurality, and a diagnosis of dementia. Improved understanding of hospice use and one-year survivorship may help LTCFs administrators, hospice care providers, and policy makers to improve hospice accessibility in this target group.

## Background

In Canada, a long-term care facility (LTCF) is “a care institution that serves diverse populations who need access to 24-hour nursing care, personal care and other therapeutic and support services” that are not provided elsewhere [1] and is a common place of death for Canadians [2–4]. Long-term care facilities are not included under the Canada Health Act [5] and are governed by provincial and territorial legislation [6]. While the majority of LTCFs are publicly funded, service delivery is provided by a mix of public (government-owned), private not-for-profit, and private for-profit providers [5, 7]. Most residents stay in LTCFs until death [2–4], making hospice care important in LTCFs.

Hospice care can reduce or relieve physical and psychological symptoms, provide comfort and dignity for the person living with the illness as well as the best quality of life for both this person and his or her family [8]. The general eligibility for hospice care program in Canada is: a) the person has a life limiting illness with a prognosis of 6 months or less, b) a decision has been made to focus on comfort rather than cure, and c) resuscitation will not be used when the illness brings a natural death [9]. In the Resident Assessment Instrument Minimum Data Set Version 2.0 (RAI-MDS 2.0) assessment, hospice care is defined as “a program for terminally ill persons where services are necessary for the palliation and management of terminal illness and related conditions” [10]. However, it can be difficult to recognize these terminal stages, particularly when the person may have several medical problems but no specific terminal diagnosis. Hospice care in Canada has traditionally been offered only in the last weeks or months of life [11]. Late referrals for hospice care limit the ability of health systems to reach maximum potential for reduction or relief of suffering and healthcare cost containment [12].

Hospice care can be provided in a variety of settings, such as homes, hospitals, LTCFs, and free-standing hospice facilities [13]. Hospitals may not be the best location for comfortable end-of-life care, as they are designed to address severe and urgent needs [13]. Providing hospice services in LTCFs is cost-efficient compared to providing hospice care at private homes or hospice facilities [14]. For example, hospice facilities may have greater revenues by increasing their resident volume, utilizing staff more efficiently, overlapping basic services, and increasing the average length of stay [15]. The basic services such as housekeeping and central supplies can be shared by hospice facilities and LTCFs. Long-term care facilities who enroll residents in a hospice care program can increase their competitiveness of the market by promising to provide hospice care to residents who are nearing the end of life, reduce in-house staff time while providing these special services, and benefit from the knowledge of hospice staff [15, 16].

While a large proportion of Canadians die in LTCFs each year, most LTCFs lack a formalized hospice care program or adequate resources to provide comprehensive end-of-life care [17, 18]. Some residents who did not meet the hospice eligibility received hospice care, while many residents who may have benefitted from hospice care did not receive it [19]. However, there is limited research examining hospice use in LTCFs in Canada [20]. To give a comprehensive understanding of hospice use and survivorship in LTC settings, this study examined the characteristics of, and one-year survivorship among, residents who received hospice care versus those who did not in LTCFs in Canada. This study was guided by the following two main questions: (a) What are the characteristics of LTCF residents in Canada who received versus who did not receive hospice care by their one-year survivorship? and (b) What variables can predict one-year survivorship of hospice use among LTCF residents?

### Conceptual framework

To better understand hospice services utilization in LTCFs in Canada, Andersen and Newman’s behavioral model [21] was used to guide this study. This model was designed to assist in explaining and predicting demographic and societal determinants for utilization of health services and identifying access disparities and other barriers to these services [21]. This behavioral model was widely used for studies on health services utilization [22, 23]. The behavioral model indicates that the utilization of health services was a result of three components of population characteristics: (a) the predisposition of the person to use services (predisposing characteristics), (b) the person’s ability to secure the services (enabling resources), and (c) the person’s illness level (needs) [21]. This model was used in this study to assist in evaluating the current hospice care practices in LTCFs in Canada and understanding how the residents’ predisposing, enabling, and need characteristics influence their utilization of hospice care services.

## Methods

### Study design and data sources

This population-based cohort study used health administrative data from the Canadian Continuing Reporting System (CCRS) and the Discharge Abstract Database (DAD) from the Canadian Institute for Health Information (CIHI). The Canadian Institute for Health Information is an independent and not-for-profit organization that provides essential information on Canada’s health systems and the health of Canadians. The Canadian Institute for Health Information has a comprehensive and high-standard Data and Information Quality Program to ensure the data can be trusted by stakeholders [24]. All persons who resided in a LTCF and who had a RAI-MDS 2.0 assessment in the CCRS database between Jan. 1st, 2015 and Dec 31st, 2015 were included in this study (*N* = 185,715). Death records were linked through the RAI-MDS 2.0 discharge form and the DAD up to Dec 31th, 2016.

The RAI-MDS 2.0 was completed by trained clinical professionals (including registered nurses, social workers, physicians) through direct observation over all shifts prior to the assessments and also included information from chart records, the resident, and his/her family (when available) [24]. The RAI-MDS 2.0 contains standardized and comprehensive information of residents receiving 24-h continuing care services in LTCFs in Canada [25]. The full assessment of the RAI-MDS 2.0 are required for each resident at admission, upon significant changes in status, and within 1 year of the last full assessment [26]. Residents are also assessed quarterly on a subset of the full assessment. This study used a mix of admission assessments (17.4%), significant change in status full assessment (5.3%), annual full assessments (22.0%), and quarterly assessments (55.3%). The Discharge Abstract Database captures information on hospital discharges (including deaths, sign-outs, and transfers) directly from acute care facilities or from their respective health/regional authority or ministry/department of health in all provinces and territories except Quebec [27].

### Measures

**Fig. 1.**
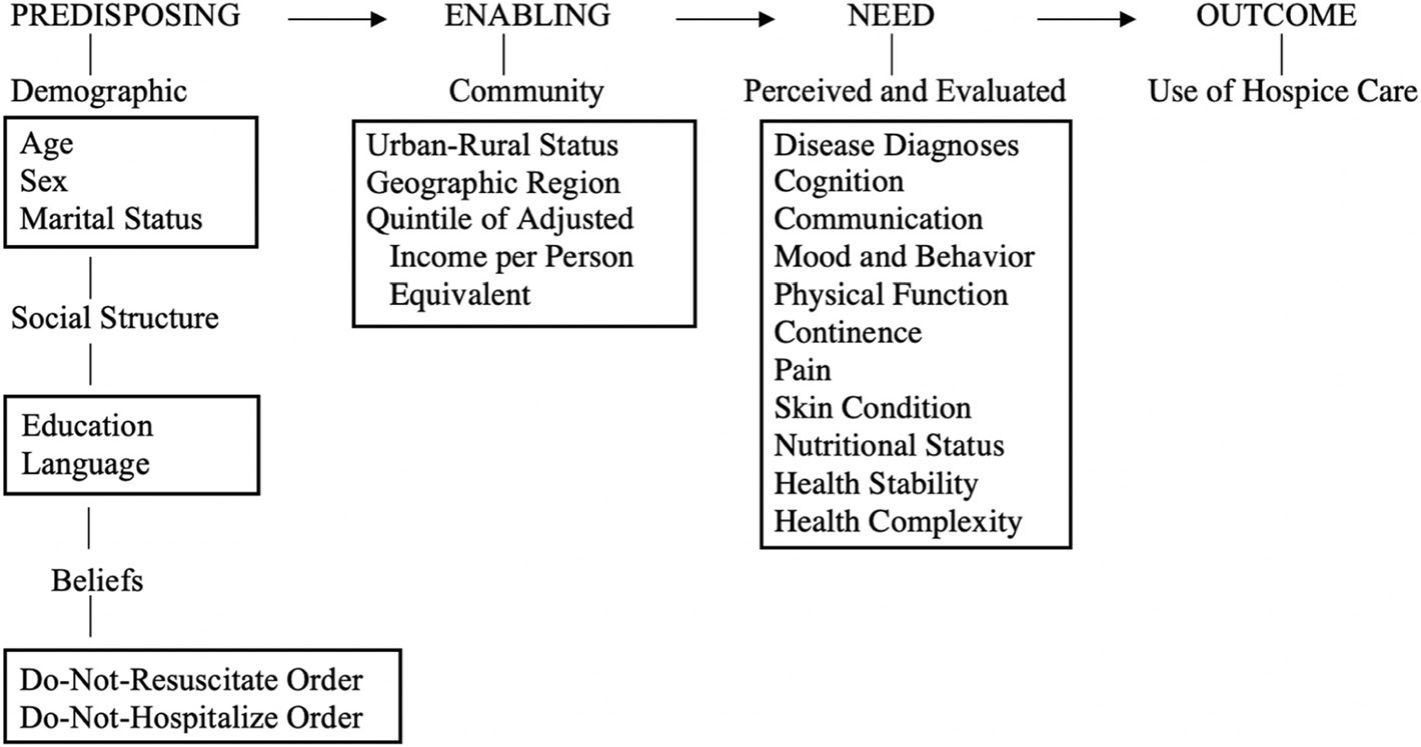
The conceptual framework of this study: a modified Andersen and Newman’s behavioral model. Adapted from “Societal and individual determinants of medical care utilization in the United States” by R. M. Andersen and J. F. Newman, 1973, Milbank Memorial Fund Quarterly, 51, 107

Based on Andersen and Newman’s (1973) behavioral model, three determinants (predisposing characteristics, enabling characteristics, and need characteristics) of hospice use in LTCF residents were examined in this study (Fig. 1).

Predisposing characteristics included age, gender, marital status, language, education, and advance directives (do-not-resuscitate [DNR] and do-not-hospitalize [DNH] orders). Age was measured in years and also categorized into five age groups.

Enabling characteristics included province, urban-rural status, and Quintile of Adjusted Income per Person Equivalent (QAIPPE). According to Statistics Canada, “an urban area was defined as having a population of at least 1,000 and a density of 400 or more people per square kilometre and all areas outside urban areas was defined as rural areas” [28]. The QAIPPE is an area-based socioeconomic measure of neighbourhood income quintile for the LTCF [29].

Need characteristics included disease diagnoses, cognitive function, communication function, mood and behavior, physical function, continence, pain, skin condition, nutritional status, health stability, and complexity of clinical needs. Risk and clinical summary outcome scales embedded within the RAI-MDS 2.0 were used to examine need characteristics. The outcome scales included the Cognitive Performance Scale (CPS) [30], the Depression Rating Scale (DRS) [31], the Index of Social Engagement (ISE) [32], the Activities of Daily Living Self-Performance Hierarchy Scale (ADL) [33], the Changes in Health, End-Stage Disease, and Signs and Symptoms (CHESS) [34], the Aggressive Behavior Scale (ABS) [35], the Pain Scale [36], and the Pressure Ulcer Risk Scale (PURS) [37] (Table 1).

**Table 1:** Selected interRAI outcome scales common to the RAI-MDS 2.0 instruments with description and ranges

Outcome scales	Description	Range
Cognitive Performance Scale (CPS)	Measures cognitive status of the residents • Validated against Mini-Mental State Examination (MMSE) (*R*^*2*^ = 0.81)	Range from 0 to 6 • 0 indicates no cognitive impairment (cognitively intact) • 6 indicates severe cognitive impairment
Depression Rating Scale (DRS)	Clinical screener for depression • Validated against Hamilton Depression Rating Scale (*r* = 0.69)	Range from 0 to 14 • Score greater than 3 indicates possible depression
Aggressive Behavior Scale (ABS)	Measures aggressive behavior • Validated against Cohen Mansfield Agitation Inventory (CMAI) (*r* = 0.72)	Range from 0 to 12 • 0 indicates no signs of aggression • 12 indicates very severe aggression
Index of Social Engagement (ISE) Scale	Measures residents’ quality of life and level of social involvement in activities within the long-term care facility. • showed reasonable internal consistency (Cronbach’s alpha = 0.72) and an acceptable construct validity (Bentler’s Fit Index > 0.98)	Range from 0 to 6 • 0 indicates no/low social engagement • 6 indicates high social engagement
Activities of Daily Living Hierarchy Scale (ADL-H)	Measures the level of impairment in the disablement process of early, mid, and late loss of activities of daily living. • Validated against Barthel Index (*R*^*2*^ = 0.74)	Range from 0 to 6 • 0 indicates no functional impairment • 6 indicates severe functional impairment
Pain Scale	Measures pain presence and intensity • Validated against the Visual Analogue Scale (*κ* = 0.71)	Range from 0 to 4 • 0 indicates no pain • 4 indicates severe pain
Pressure Ulcer Risk Scale (PURS)	Measures pressure ulcer risk • Verified to be a good predictor of pressure ulcer risk (c statistics = 0.708) and validated against the Braden Scale (*r* = 0.66)	Range from 0 to 8 • 0 indicates no risk for pressure ulcers • 8 indicates severe risk for pressure ulcers
Changes in Health End Stage Signs and Symptoms (CHESS) Scale	Measure of health instability as a clinical outcome and predictor of mortality • Verified to be a good predictor of mortality (*p* < 0.0001)	Range from 0 to 5 • 0 indicates no health instability • 5 indicates very high health instability

Clinical Assessment Protocols, embedded in the RAI-MDS 2.0, were also used to assess need characteristics. The CAPs “identify areas in which a resident has a higher than expected rate of decline, an increased potential to improve, and symptoms that could be alleviated if a problem was addressed” [38]. There are 19 CAPs including activities of daily living, physical restraints, cognitive loss, delirium, communication, mood, behaviour, activities, social relationship, falls, pain, pressure ulcer, cardio-respiratory conditions, undernutrition, dehydration, feeding tube, appropriate medications, urinary incontinence, and bowel conditions [39]. The number of triggered CAPs was used to identify complexity of clinical needs in this study [40].

### Statistical analyses

Analyses were conducted using SAS software for Windows Version 9.4, Cary, NC: SAS institute, Inc. At stage 1, univariate analyses including means, medians, standard deviations, and percentages were used to describe the characteristics of residents. At stage 2, bivariate analyses including t-tests for continuous variables and chi-square test for categorical variables were performed to test the statistically significant differences in characteristics of residents in four groups: a) did not receive hospice care and were alive 1 year following the assessment, b) did not receive hospice care and died within 1 year, c) received hospice care and were alive 1 year following the assessment, and d) received hospice care and died within 1 year. At stage 3, multinomial logistic regression analysis was conducted to explain how the residents’ characteristics influence their hospice use with residents who did not receive hospice care and were alive 1 year following the assessment as the reference group.

Variables that had more than 5% of missing values were not included in the multinomial logistic regression analysis [41]. For variables with less than 5% of missing values, complete case analysis was applied for the predictive modelling. To reduce multicollinearity, variables that were already in the outcome scales were not considered for the predictive model building. Multicollinearity was measured by variance inflation factors (VIF), tolerance, and condition index. Multicollinearity was thought to be present when VIF value exceeded 4.0, tolerance was less than 0.2, or condition index exceeded 30.0 [42]. All statistical tests were based on two-sided probability and an alpha of 0.05 or less was used to indicate statistical significance [41].

### Ethical considerations

Data access was granted and monitored by CIHI. Due to the use of de-identified secondary data, the University of Northern British Columbia ethics board confirmed ethics approval for this project was not required.

## Results

### Sample characteristics

**Fig. 2.**
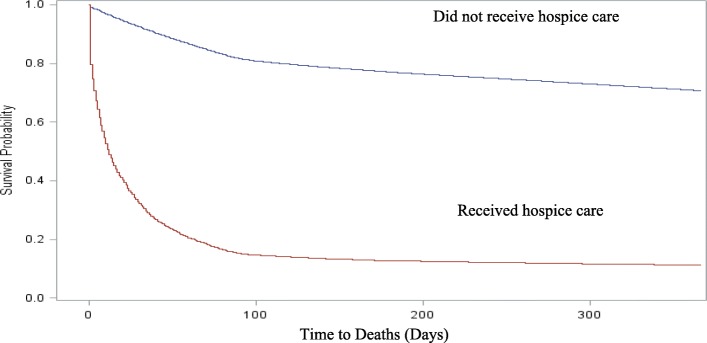
Survival analysis by hospice use in long-term care facilities in Canada in 2015 (*N* = 185,715)

There were 185,715 unique residents living in LTCFs assessed with the RAI-MDS 2.0 assessment in Canada in 2015. Residents in LTCFs in Canada had a mean age of 83.0 years (±11.4 years). More than half were aged over 85 years (54.3%) and widowed (51.6%). Two thirds were female (65.8%). Most lived in an urban LTCF (85.2%) (Table 2). One third died within 1 year (30.9%, *n* = 57,398/185,715), most of whom died within 3 months (65.5%, *n* = 37,602/57,398) (Fig. 2). Of all 185,715 residents in LTCFs in Canada in 2015, only 2.7% of the residents received hospice care (*n* = 4973/185,715). Of those who received hospice care, 88.9% died within 1 year (*n* = 4417/4973), while about 10.1% were still alive 1 year following the assessment (*n* = 556/4973). Of those who did not receive hospice care, 29.3% died within 1 year (*n* = 52,981/180,742).

**Table 2:** Characteristics of residents in long-term care facilities in Canada at baseline in 2015 (*N* = 185,715)

Variables	Total population, *N* = 185,715	Did not receive hospice care		Received hospice care		*p*-value
		Alive after 1 year, 68.8%, *N* = 127,761	Died within 1 year, 28.5%, *N* = 52,981	Alive after 1 year, 0.3%, *N* = 556	Died within 1 year, 2.4%, *N* = 4417	
Determinants 1: Predisposing Characteristics						
Age Group (in years)						< 0.0001
19–64	7.6 (14,055)	9.2 (11,790)	3.0 (1574)	15.1 (84)	13.7 (607)	
65–74	11.0 (20,509)	12.7 (161,66)	6.7 (3535)	17.1 (95)	16.1 (713)	
75–84	27.2 (50,483)	28.6 (36,490)	23.9 (12,647)	28.6 (136)	23. 9 (1210)	
85–94	43.9 (81,435)	41.4 (52,922)	50.6 (26,814)	33.6 (187)	34.2 (1512)	
95+	10.4 (19,233)	8.1 (10,393)	15.9 (8411)	9.7 (54)	8.5 (375)	
Female Sex	65.8 (122,145)	66.8 (85,289)	64.2 (33,963)	60.3 (335)	57.9 (2558)	< 0.0001
Marital Status						< 0.0001
Never Married	10.3 (16,647)	11.6 (13,129)	6.9 (3069)	8.3 (42)	10.3 (407)	
Married	27.5 (44,546)	27.1 (30,637)	27.2 (12,122)	36.9 (187)	40.5 (1600)	
Widowed	51.6 (83,583)	49.7 (56,137)	57.4 (25,612)	44.0 (233)	40.7 (1611)	
Separated	3.1 (5017)	3.3 (3683)	2.8 (1238)	2.2 (11)	2.2 (85)	
Divorced	7.6 (12,337)	8.4 (9478)	5.8 (2564)	8.7 (44)	6.4 (251)	
Primary Language Spoken	83.6 (155,309)	82.5 (105,345)	86.3 (45,694)	83.6 (465)	86.1 (3805)	< 0.0001
English	2.5 (4560)	2.5 (3245)	2.1 (1112)	4.9 (27)	4.0 (176)	
French	13.9 (25,846)	15.0 (19,171)	11.7 (6175)	11.5 (64)	9.9 (436)	
Education						< 0.0001
8th Grade or Less	28.5 (33,527)	28.6 (23,158)	28.5 (9877)	28.1 (62)	25.6 (430)	
9th Grade-High School	44.0 (36,767)	43.9 (35,522)	44.5 (15,428)	37.1 (82)	43.2 (725)	
Technical/Trade School/College	16.7 (19,666)	16.8 (13,608)	16.5 (5704	22.6 (50)	18.1 (304)	
Bachelor’s or Higher Degree	10.7 (12,596)	10.7 (8694)	10.5 (3654)	12.2 (27)	13.2 (221)	
Advance Directives						< 0.0001
Do Not Resuscitate	89.8 (137,001)	76.6 (92,034)	89.4 (44,946)	92.5 (457)	89.4 (3614)	
Do Not Hospitalize	35.7 (62,244)	30.7 (36,710)	45.4 (22,753)	58.0 (282)	62.7 (2499)	
Determinants 2: Enabling Characteristics						
Province						< 0.0001
Alberta	10.0 (18,505)	8.8 (11,236)	13.4 (7097)	1.3 (7)	3.7 (165)	
British Columbia	16.3 (30,288)	15.3 (19,494)	20.2 (10,695)	3.2 (18)	1.8 (81)	
Ontario	62.8 (116,552)	65.8 (84,114)	52.5 (27,796)	92.8 (516)	93.4 (4126)	
Other Provinces	11.0 (20,370)	10.2 (12,917)	14.1 (7393)	2.7 (15)	1.1 (45)	
Urban Facility	85.2 (158,246)	86.0 (109,860)	82.6 (43,715)	93.2 (518)	940 (4153)	< 0.0001
Facility Neighbourhood Income Quintile						< 0.0001
Lowest (QAIPPE = 1)	26.1 (48,213)	26.4 (33,511)	26.4 (13,928)	15.5 (86)	15.6 (688)	
Low (QAIPPE = 2)	19.1 (35,280)	18.9 (24,016)	18.8 (9901)	25.2 (140)	27.8 (1233)	
Medium (QAIPPE = 3)	20.4 (37,655)	20.4 (25,833)	20.7 (10,893)	18.2 (101)	18.8 (828)	
High (QAIPPE = 4)	18.9 (34912)	19.1 (24,214)	18.4 (9691)	29.0 (161)	19.2 (846)	
Highest (QAIPPE = 5)	15.4 (28,497)	15.2 (19,293)	15.8 (8320)	12.1 (67)	18.6 (817)	
Determinants 3: Need Characteristics						
Change in Cognitive Status						< 0.0001
No Change	85.9 (159,103)	90.2 (115,121)	79.6 (41,951)	69.2 (384)	38.9 (1647)	
Improved	1.8 (3349)	2.0 (2515)	1.5 (779)	2.2 (12)	1.0 (43)	
Deteriorated	12.3 (22,683)	7.8 (9985)	19.0 (9995)	28.7 (159)	60.1 (2544)	
Cognition						< 0.0001
No-Mild Cognitive Impairment (CPS = 0–1)	22.3 (41,329)	25.6 (32,689)	14.3 (7552)	29.1 (162)	21.0 (926)	
Moderate Cognitive Impairment (CPS = 2–4)	55.5 (103,097)	56.9 (72,660)	53.3 (28,240)	47.8 (266)	43.7 (1931)	
Severe Cognitive Impairment (CPS = 5–6)	22.2 (41,289)	17.5 (22,412)	32.4 (17,189)	23.0 (128)	35.3 (1560)	
Change in Communication						< 0.0001
No Change	91.9 (170,042)	95.1 (121,307)	86.3 (45,737)	83.4 (462)	59.9 (2535)	
Improved	0.7 (1212)	0.7 (918)	0.5 (272)	1.3 (7)	0.4 (15)	
Deteriorated	7.5 (13,881)	4.2 (5396)	12.7 (6716)	15.3 (85)	39.8 (1684)	
Change in Mood						< 0.0001
No Change	82.4 (152,576)	85.0 (108,505)	78.9 (41,592)	66.2 (368)	49.9 (2111)	
Improved	5.1 (9465)	5.3 (6772)	4.8 (2518)	4.7 (26)	3.5 (149)	
Deteriorated	12.5 (23,094)	9.7 (12,344)	16.3 (8615)	29.0 (161)	46.6 (1974)	
Depression						< 0.0001
No Depressive Symptoms (DRS = 0)	41.0 (75,838)	43.2 (55,161)	36.1 (19,043)	41.6 (231)	33.1 (1403)	
Some Depressive Symptoms (DRS = 1–2)	30.3 (56,163)	29.5 (37,633)	32.2 (16,996)	28.7 (159)	33.2 (1405)	
Mild Depressive Disorder (DRS = 3–5)	19.8 (36,669)	19.1 (24,364)	21.1 (11,129)	23.4 (130)	24.7 (1046)	
Moderate-Severe Depressive Disorder (DRS = 6–14)	8.9 (16,465)	8.2 (10,463)	10.6 (5587)	6.3 (35)	9.0 (380)	
Change in Behavior Symptom						< 0.0001
No Change	85.0 (157,437)	87.3 (111,368)	81.2 (43,796)	78.2 (434)	67.1 (2839)	
Improved	4.4 (8181)	4.3 (5487)	4.7 (2486)	6.0 (33)	4.1 (175)	
Deteriorated	10.5 (19,517)	8.4 (10,766)	14.1 (7443)	15.9 (88)	28.8 (1220)	
Aggressive Behavior						< 0.0001
No Signs of Aggression (ABS = 0)	58.9 (108,962)	60.8 (77,594)	53.8 (28,345)	68.2 (379)	62.5 (2644)	
Mild to Moderate Aggression (ABS = 1–4)	33.0 (61,091)	31.6 (40,337)	36.4 (19,209)	27.9 (155)	32.8 (1390)	
Severe Aggression (ABS = 5–12)	8.2 (15,082)	7.6 (9690)	9.8 (5171)	3.8 (21)	4.7 (200)	
Social Engagement						< 0.0001
No to Low Social Engagement (ISE = 0–1)	27.3 (50,606)	21.4 (27,303)	39.0 (20,645)	36.9 (205)	55.5 (2453)	
Moderate Social Engagement (ISE = 2–4)	52.5 (97,572)	54.4 (69,529)	49.4 (26,177)	46.0 (256)	36.5 (1610)	
High Social Engagement (ISE = 5–6)	20.2 (37,537)	24.2 (88,883)	11.6 (6159)	17.1 (95)	8.0 (354)	
Change in Physical Function						< 0.0001
No Change	74.6 (138,594)	79.2 (101,127)	68.6 (169,087)	46.8 (260)	19.3 (854)	
Improved	4.5 (8382)	5.6 (7130)	2.2 (1184)	5.9 (33)	0.8 (35)	
Deteriorated	20.9 (38,739)	15.3 (19,504)	29.2 (15,444)	47.3 (263)	79.9 (3528)	
Physical Function						< 0.0001
No Functional Impairment (ADL-H = 0)	4.2 (7774)	5.3 (6817)	1.8 (927)	3.6 (20)	0.2 (10)	
Mild Functional Impairment (ADL-H = 1–2)	16.7 (31,052)	20.3 (25,930)	9.1 (4836)	15.1 (84)	4.6 (202)	
Moderate Functional Impairment (ADL-H = 3–4)	44.4 (82,424)	47.5 (60,706)	39.2 (20,755)	29.7 (165)	18.1 (798)	
Severe Functional Impairment (ADL-H = 5–6)	34.7 (64,465)	26.9 (34,308)	50.0 (26,463)	51.6 (287)	77.1 (3407)	
Bowel Continence						< 0.0001
Continent	34.9 (64,768)	40.9 (52,279)	21.8 (11,545)	31.7 (176)	17.4 (768)	
Usually Continent	11.8 (21,915)	12.3 (15,712)	10.8 (5745)	10.1 (56)	9.1 (402)	
Occasionally continent	8.0 (14,876)	8.0 (10,161)	8.4 (4425)	5.2 (29)	5.9 (261)	
Frequently Continent	15.0 (27,873)	14.2 (18,177)	17.1 (9036)	11.2 (62)	13.5 (598)	
Incontinent	30.3 (27,873)	24.6 (31,432)	42.0 (22,230)	41.9 (233)	54.1 (2388)	
Bladder Continence						< 0.0001
Continent	20.7 (38,469)	22.9 (29,197)	14.2 (7512)	38.5 (214)	35.0 (1546)	
Usually Continent	8.6 (15,941)	9.6 (12,304)	6.3 (3310)	8.1 (45)	6.4 (262)	
Occasionally continent	9.9 (18,317)	10.8 (13,795)	7.8 (4149)	7.0 (39)	7.6 (334)	
Frequently Continent	24.5 (45,548)	25.6 (32,656)	23.1 (12,234)	13.1 (73)	13.2 (585)	
Incontinent	36.3 (67,440)	31.2 (39,809)	48.7 (25,776)	33.3 (185)	37.8 (1670)	
Change in Urinary Continence						< 0.0001
No Change	85.8 (159,362)	88.2 (112,711)	83.1 (44,026)	71.2 (396)	50.5 (2229)	
Improved	2.8 (5199)	3.1 (3892)	2.0 (1083)	5.2 (29)	4.4 (195)	
Deteriorated	11.4 (21,154)	8.7 (11,158)	14.9 (7872)	23.6 (131)	45.1 (1993)	
Pain Symptoms Frequency						< 0.0001
No Pain	60.5 (112,389)	63.5 (81,083)	56.8 (30,110)	35.8 (199)	22.6 (997)	
Pain Less Than Daily	24.8 (45,980)	23.8 (30,391)	25.9 (13,702)	34.2 (190)	38.4 (1697)	
Pain Daily	14.7 (27,346)	12.8 (16,287)	17.3 (9169)	30.0 (167)	39.0 (1723)	
Pain Symptoms Intensity						< 0.0001
Mild Pain	45.7 (33,523)	48.6 (22,702)	42.9 (9.813)	32.2 (115)	26.1 (893)	
Moderate Pain	46.1 (33,791)	44.5 (20,781)	47.6 (10,880)	54.6 (195)	56.6 (1935)	
Severe Pain	8.2 (6012)	6.8 (3195)	9.5 (2178)	13.2 (47)	17.3 (593)	
Pain						< 0.0001
No Pain (Pain Scale = 0)	60.5 (112,389)	63.5 (81,083)	56.8 (30,110)	35.8 (199)	22.6 (997)	
Mild-Moderate Pain (Pain Scale = 1–2)	37.2 (68,997)	34.9 (44,540)	40.1 (21,219)	57.2 (318)	66.1 (2920)	
Severe Pain (Pain Scale = 3)	2.3 (4329)	1.7 (2138)	3.1 (1652)	7.0 (39)	11.3 (500)	
Weight Loss	11.0 (18,973)	7.3 (8719)	17.7 (8838)	28.3 (128)	40.4 (1288)	< 0.0001
Leaves Food Uneaten	33.3 (61,836)	26.0 (33,250)	47.0 (24,911)	50.9 (283)	76.8 (3392)	< 0.0001
Pressure Ulcer Risk						< 0.0001
No Pressure Ulcer Risk (PURS = 0)	19.0 (34,861)	23.8 (30,126)	8.8 (4595)	9.2 (51)	2.0 (89)	
Mild Pressure Ulcer Risk (PURS = 1–2)	33.1 (60,827)	36.5 (46,254)	26.5 (13,858)	27.9 (154)	12.8 (561)	
Moderate Pressure Ulcer Risk (PURS = 3)	31.0 (57,005)	28.(36,406)	36.8 (19,276)	30.4 (168)	26.3 (1155)	
High Pressure Ulcer Risk (PURS = 4–5)	15.4 (28,401)	10.1 (12,797)	25.1 (13,152)	30.2 (167)	51.9 (2285)	
Very High Pressure Ulcer Risk (PURS = 6–8)	1.6 (2847)	0.8 (1041)	2.8 (1483)	2.4 (13)	7.1 (310)	
Health Instability						< 0.0001
No Indication of Health Instability (CHESS = 0)	40.7 (75,639)	48.1 (61,422)	26.7 (14,127)	10.4 (58)	0.7 (32)	
Mild Health Instability (CHESS = 1–2)	47.0 (87,227)	46.4 (59,239)	51.4 (27,228)	39.0 (217)	12.3 (12.3)	
Moderate-Severe Health Instability (CHESS = 3–5)	12.3 (22,849)	5.6 (7100)	21.9 (11,626)	50.5 (281)	87.0 (3842)	
Number of CAPs Triggered						< 0.0001
0	0.7 (1228)	0.6 (763)	0.8 (434)	1.4 (8)	0.5 (23)	
1–5	51.9 (96,344)	54.7 (69,938)	46.4 (24,579)	45.5 (253)	35.6 (1574)	
6–10	45.5 (84,412)	43.2 (55,197)	49.8 (26,387)	49.8 (277)	57.8 (2551)	
11–16	1.6 (3009)	1.1 (1456)	2.4 (1266)	3.2 (18)	6.1 (269)	

*Other provinces include Manitoba, Saskatchewan, Newfoundland and Labrador, Nova Scotia, and Yukon. QAIPPE* denotes Quintile of Adjusted Income per Person Equivalent, *CPS* denotes Cognitive Performance Scale, *DRS* denotes Depression Rating Scale, *ABS* denotes Aggressive Behavior Scale, *ISE* denotes Index of Social Engagement, *ADL-H* denotes Activities of Daily Living Hierarchy, *PURS* denotes Pressure Ulcer Risk Scale, *CHESS* denotes Changes in Health, End-Stage Disease, Signs, and Symptoms Scale

#### Did not receive hospice care and alive

**Fig. 3.**
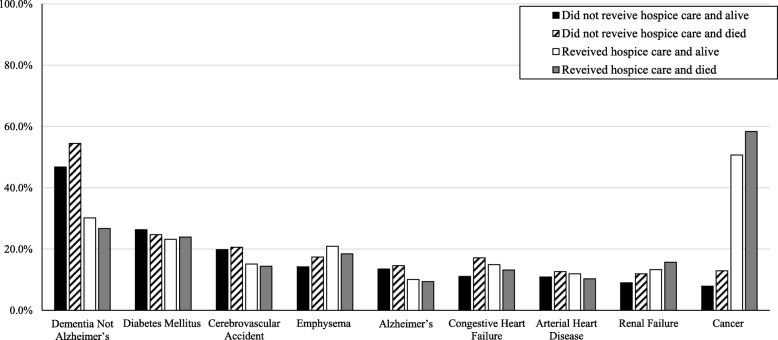
Selected common diagnoses by hospice use in long-term care facilities in Canada in 2015 (*N* = 185,715)

**Fig. 4.**
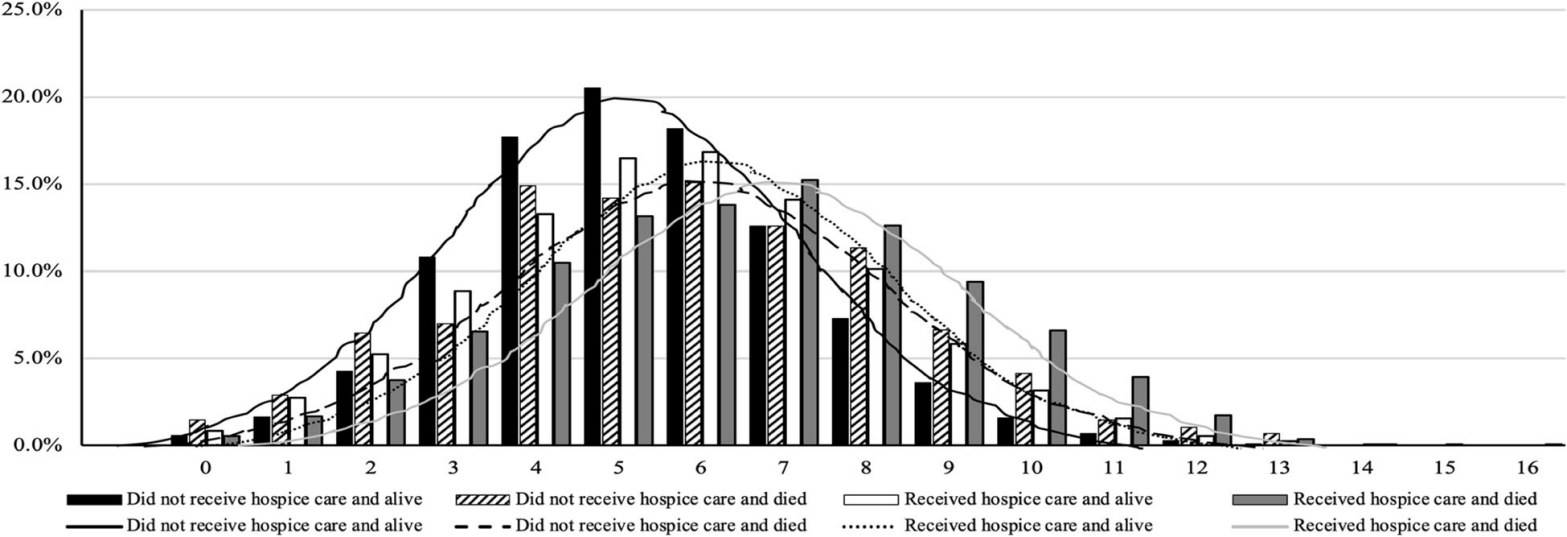
Number of clinical protocol assessments triggered by one-year survivorship of hospice use (*N* = 185,715)

Residents who did not receive hospice care and were alive 1 year following the assessment (*n* = 127,761) had a mean age of 81.7 (+ 11.8) years. Half were aged over 85 years (49.5%) and widowed (49.7%). Two thirds were female (66.8%). Three in four had a DNR order (76.6%), and less than one third had a DNH order (30.7%). The majority lived in an urban LTCF (86.0%) (Table 2). A diagnosis of dementia (46.8%) was more common than a diagnosis of cancer (7.9%) in this group (Fig. 3). The majority of the residents in this group exhibited no to moderate cognitive impairment (CPS ≤ 4, 82.5%), no to mild depressive symptoms (DRS ≤ 2, 72.7%), no signs of aggression (ABS = 0, 60.8%), moderate to high social engagement (ISE = 2–6, 78.6%), mild to moderate physical impairment (ADL-H = 1–4, 73.1%), complete bowel incontinence (24.6%), complete bladder incontinence (31.2%), no pain (Pain Scale = 0, 63.5%), no to moderate pressure risk (PURS ≤3, 89.1%), and no to mild health instability (CHESS ≤2, 94.5%) (Table 2). Less than half triggered more than five CAPs (44.7%) (Fig. 4); the top three triggered CAPs were activities of daily living (84.3%), urinary incontinence (82.5%), and mood (56.7%).

#### Did not receive hospice care and died

Residents who did not receive hospice care but died within 1 year (*n* = 52,981) had a mean age of 79.4 (+ 13.5) years. Two thirds were aged over 85 years (66.5%). More than half were widowed (57.4%). Less than two thirds were female (64.2%). The majority had a DNR order (89.4%), but less than half had a DNH order (45.4%). Most lived in an urban LTCF (82.6%) (Table 2). Dementia was a common diagnosis in this group (54.5%), but cancer was not (12.9%) (Fig. 3). About one third of the residents in this group exhibited severe cognitive impairment (CPS ≥ 5, 32.4%) and possible depressive disorder (DRS ≥ 3, 31.7%), about half had signs of aggression (ABS ≥1, 46.3%), more than one third had no to low social engagement (ISE ≤ 1, 39.0%), half had severe physical impairment (ADL-H ≥ 5, 50.0%), less than half had complete bowel incontinence (42.0%), about half had complete bladder incontinence (48.7%), less than half had mild to severe pain (Pain Scale ≥1, 43.2%), one in four had high to very high pressure risk (PURS ≥4, 27.9%), and one in five had moderate to severe health instability (CHESS ≥3, 21.9%) (Table 2). More than half had more than five CAPs triggered (52.2%) (Fig. 4); the top three triggered CAPs were urinary incontinence (77.0%), mood (58.3%), and social relationship (52.3%).

#### Received hospice care and alive

Residents who received hospice care and were alive 1 year following the assessment (*n* = 556) had a mean age of 86.5 (+ 9.2) years. Less than half were aged over 85 years (43.3%) and widowed (44.0%). The majority had a DNR order (92.5%), and more than half had a DNH order (58.0%). The majority lived in an urban LTCF (93.2%) (Table 2). A diagnosis of cancer (50.7%) was more common than a diagnosis of dementia (30.2%) in this group (Fig. 3). One fourth of the residents in this group exhibited severe cognitive impairment (CPS ≥ 5, 23.0%), less than one third had depressive disorder (DRS ≥ 3, 29.7%) and signs of aggression (ABS ≥1, 31.7%), more than one third had no to low social engagement (ISE ≤ 1, 36.9%), more than half had severe physical impairment (ADL-H ≥ 5, 51.6%), two in five had complete bowel incontinence (41.9%), one third had complete bladder incontinence (33.3%), two thirds had mild to severe pain (Pain Scale ≥1, 64.2%), one third had high to very high pressure risk (PURS ≥4, 32.6%), and half had moderate to severe health instability (CHESS ≥3, 50.5%) (Table 2). More than half had more than five CAPs triggered (53.0%) (Fig. 4), and the top three triggered CAPs were activities of daily living (68.6%), urinary incontinence (67.6%), and mood (63.6%).

#### Received hospice care and died

Residents who received hospice care and died within 1 year (*n* = 4417) had a mean age of 79.7 (+ 12.7) years. Less than half were aged 85 years and older (42.7%). More than one third were widowed (40.7%). More than half were female (57.9%). The majority had a DNR order (89.4%), and less than two thirds had a DNH order (62.7%). The majority lived in an urban LTCF (94.0%) (Table 2). More than half had a diagnosis of cancer (58.4%) and about one in five had a diagnosis of dementia (26.7%) (Fig. 3). One third of the residents in this group exhibited severe cognitive impairment (CPS ≥ 5, 35.3%) and possible depressive disorder (DRS ≥ 3, 33.7%), more than one third had signs of aggression (ABS ≥1, 37.5%), more than half had no to low social engagement (ISE ≤ 1, 55.5%), more than two thirds had severe physical impairment (ADL-H ≥ 5, 77.1%), more than half had complete bowel incontinence (54.1%), more than one third had complete bladder incontinence (37.8%), more than two thirds had mild to severe pain (Pain Scale ≥1, 77.4%), more than half had high pressure ulcer risk (PURS ≥4, 59.0%), and most had moderate to severe health instability (CHESS ≥3, 87.0%) (Table 2). Two thirds triggered more than five CAPs (63.9%) (Fig. 4), and the top three triggered CAPs were urinary incontinence (64.7%), mood (64.1%), and social relationship (48.6%).

### Differences among four groups

Compared to residents who did not receive hospice care and were alive 1 year following the assessment, residents who did not receive hospice care but died within 1 year (*n* = 556) were older (66.5% aged over 85 years vs. 49.5%), had a higher percentage of being widowed (57.4% vs. 49.7%), having a DNR order (89.4% vs. 76.6%), and having a DNH order (45.4% vs. 30.7%) (Table 2), but a lower percentage of living in an urban LTCF (82.6% vs 86.0%). Similar to those who did not receive hospice care and were alive 1 year following the assessment, dementia was a common diagnosis in this group (54.5%) but cancer was not (12.9%) (Fig. 3). Residents who did not receive hospice care but died within 1 year had more severe and complex clinical needs than those who did not receive hospice care and were alive 1 year following the assessment (Table 2; Fig. 4).

Compared to residents who did not receive hospice care and were alive 1 year following the assessment, residents who received hospice care but were alive 1 year following the assessment were younger (43.3% aged over 75 years vs. 49.5%), had a lower percentage of being widowed (44.0% vs. 49.7%), but a higher percentage of having a DNR order (92.5% vs. 76.6%), and having a DNH order (58.0% vs. 30.7%), and living in an urban LTCF (93.2% vs 86.0%) (Table 2). Cancer was more common in this group (50.7% vs 7.9%), while dementia was less common (30.2% vs 46.8%) (Fig. 3). This group exhibited more severe acute clinical needs than those who did not receive hospice care but died within 1 year of assessment with regard to mild to severe pain (Pain Scale ≥1, 64.2% vs. 43.2%), high to very high pressure risk (PURS ≥4, 32.6% vs. 27.9%), and moderate to severe health instability (CHESS ≥3, 50.5% vs. 21.9%), but had less chronic clinical needs in terms of severe cognitive impairment (CPS ≥ 5; 23.0% vs. 32.4%) and bladder incontinence (33.3% vs. 48.7%) (Table 2).

Compared to residents who did not receive hospice care and were alive 1 year following the assessment, residents who received hospice care and died within 1 year were younger (42.7% aged over 75 years vs. 49.5%), had a lower percentage of being widowed (40.7% vs. 49.7%), but a higher percentage of having a DNR order (89.4% vs. 76.6%), having a DNH order (62.7% vs. 30.7%), and living in an urban LTCF (94.0% vs. 86.0%) (Table 2). Compared to the other three groups, this group had the highest percentage of cancer diagnosis (58.4%) and the most severe and complex clinical conditions with respect to cognition, depression, social engagement, physical function, bowel control, pain, pressure ulcer risk, health instability and highest complexity (Table 2, Figs. 3, 4).

### Multivariate results

As shown in Table 3, compared to residents who did not receive hospice care and were alive 1 year following the assessment, residents who did not receive hospice care but died within 1 year were less like to live in an urban LTCF (OR = 0.78, 95% CI = 0.75–0.80), and were more likely to be older (OR range from 1.60 [95% CI = 1.50–1.72] among 65–74 age group to 5.45 [95% CI = 5.11–5.82] among 95+ age group) and have moderate (OR = 1.21, 95% CI = 1.11–2.33) to severe (OR = 1.83, 95% CI = 1.67–2.01) physical impairment, have mild-moderate (OR = 1.13, 95% CI = 1.10–1.16) to severe (OR = 1.39, 95% CI = 1.27–1.53) pain, pressure ulcer risk (OR range from 1.38 [95% CI = 1.31–1.44] in mild pressure ulcer risk category to 2.64 [95% CI = 2.36–2.97] in very high pressure ulcer risk category) and mild (OR = 1.55, 95% CI = 1.50–1.59) to moderate-severe (OR = 3.53, 95% CI = 3.34–3.71) health instability.

**Table 3:** Multinomial logistic regression on 1-year survivorship of hospice use (odds ratios and 95% confidence intervals)

Variables	Did not receive hospice	Received hospice care	
	Died within 1 year, 28.5%, *N* = 52,981	Alive after 1 year, 0.3%, *N* = 556	Died within 1 year, 2.4%, *N* = 4417
Age Group (ref = 19–64)			
65–74	1.60 (1.50–1.72)****	0.86 (0.64–1.17)	0.93 (0.79–1.09)
75–84	2.39 (2.25–2.54)****	0.63 (0.47–0.84)**	0.86 (0.74–0.99)*
85–94	3.46 (3.26–3.67)****	0.66 (0.50–0.88)**	0.89 (0.78–1.02)
95+	5.45 (5.11–5.82)****	1.06 (0.74–1.53)	1.31 (1.10–1.56)**
Urban-Rural Status (ref = Rural)			
Urban	0.78 (0.75–0.80)****	2.04 (1.46–2.84)**	2.17 (1.89–2.50)****
Dementia Not Alzheimer’s (ref = No)			
Yes	1.04 (1.01–1.07)*	0.71 (0.57–0.87)*	0.59 (0.54–0.65)****
Cancer (ref = No)			
Yes	1.68 (1.62–1.74)****	7.98 (6.68–9.54)****	8.96 (8.27–9.71)****
Cognition (ref = No-Mild Cognitive Impairment [CPS = 0–1])			
Moderate Cognitive Impairment (CPS = 2–4)	1.10 (1.06–1.15)****	1.08 (0.81–1.45)	0.91 (0.78–1.05)
Severe Cognitive Impairment (CPS = 5–6)	1.33 (1.27–1.40)****	1.41 (0.97–2.06)	1.27 (1.06–1.52)**
Depression (ref = No Depressive Symptoms [DRS = 0])			
Some Depressive Symptoms (DRS = 1–2)	1.04 (1.00–1.07)*	0.87 (0.67–1.14)	0.81 (0.71–0.92)***
Mild Depressive Disorder (DRS = 3–5)	1.01 (0.97–1.05)	1.18 (0.87–1.61)	0.89 (0.77–1.02)
Moderate-Severe Depressive Disorder (DRS = 6–14)	1.03 (0.98–1.09)	0.74 (0.46–1.18)	0.72 (0.60–0.87)***
Aggressive Behavior (ref = No Signs of Aggression [ABS = 0])			
Mild to Moderate Aggression (ABS = 1–4)	0.95 (0.92–0.98)**	0.66 (0.51–0.85)**	0.80 (0.71–0.89)****
Severe Aggression (ABS = 5–12)	0.90 (0.86–0.95)***	0.36 (0.21–0.62)***	0.49 (0.39–0.60)****
Social Engagement (ref = No-Low Social Engagement [ISE = 0–1])			
Moderate Social Engagement (ISE = 2–4)	0.74 (0.72–0.76)****	0.67 (0.52–0.86)**	0.52 (0.46–0.58)****
High Social Engagement (ISE = 5–6)	0.54 (0.52–0.57)****	0.63 (0.45–0.90)*	0.39 (0.33–0.48)****
Physical Function (ref = No Functional Impairment [ADL-H = 0])			
Mild Functional Impairment (ADL-H = 1–2)	1.05 (0.96–1.15)	0.74 (0.38–1.45)	1.85 (0.72–4.73)
Moderate Functional Impairment (ADL-H = 3–4)	1.21 (1.11–1.33)****	0.48 (0.25–0.95)*	1.84 (0.73–4.65)
Severe Functional Impairment (ADL-H = 5–6)	1.83 (1.67–2.01)****	0.86 (0.43–1.72)	5.38 (2.12–13.64)***
Pain (ref = No Pain [Pain Scale = 0])			
Mild-Moderate Pain (Pain Scale = 1–2)	1.13 (1.10–1.16)****	1.29 (1.02–1.63)*	2.03 (1.82–2.28)****
Severe Pain (Pain Scale = 3)	1.39 (1.27–1.53)****	1.33 (0.79–2.24)	2.68 (2.19–3.27)****
Pressure Ulcer Risk (ref = No Pressure Ulcer Risk [PURS = 0])			
Mild Pressure Ulcer Risk (PURS = 1–2)	1.38 (1.31–1.44)****	1.33 (0.84–2.12)	1.23 (0.82–1.84)
Moderate Pressure Ulcer Risk (PURS = 3)	1.69 (1.60–1.78)****	2.07 (1.25–3.44)**	2.18 (1.45–3.29)***
High Pressure Ulcer Risk (PURS = 4–5)	2.30 (2.17–2.45)****	2.18 (1.27–3.75)**	3.05 (2.02–4.61)****
Very High Pressure Ulcer Risk (PURS = 6–8)	2.64 (2.36–2.97)****	1.21 (0.50–2.92)	3.50 (2.24–4.38)****
Health Instability (ref = No Indication of Health Instability [CHESS = 0])			
Mild Health Instability (CHESS = 1–2)	1.55 (1.50–1.59)****	2.83 (2.01–3.98)****	10.40 (6.67–16.22)****
Moderate-Severe Health Instability (CHESS = 3–5)	3.53 (3.34–3.71)****	17.00 (15.60–24.88)****	205.37 (132.09–319.30)****

Wald χ^2^(12, *N* = 182,858) = 33,066.2, *p* < 0.0001, AIC = 212,328.1, classification rate = 73.4%

Reference group is residents who did not receive hospice care but were alive 1 year following the assessment

*CPS* denotes Cognitive Performance Scale, *DRS* denotes Depression Rating Scale, *ABS* denotes Aggressive Behavior Scale, *ISE* denotes Index of Social Engagement, *ADL-H* denotes Activities of Daily Living Hierarchy, *PURS* denotes Pressure Ulcer Risk Scale, *CHESS* denotes Changes in Health, End-Stage Disease, Signs, and Symptoms Scale

*p < 0 .05, **p < 0 .01, ***p < 0.001, ****p < 0.0001

Compared to residents who did not receive hospice care and were alive 1 year following the assessment, residents who received hospice care but were alive 1 year following the assessment were more likely to be aged between 75 to 84 (OR = 0.63, 95% CI = 0.47–0.84) and 85 to 94 (OR = 0.66, 95% CI = 0.50–0.88) years, live in urban LTCF (OR = 2.04, 95% CI = 1.46–2.84) and have a diagnosis of cancer (OR = 7.98, 95% CI = 6.68–9.54), moderate (OR = 2.07, 95% CI = 1.25–3.44) to high (OR = 2.18, 95% CI = 1.27–3.75) pressure ulcer risk, and mild (OR = 2.83, 95% CI = 2.01–3.98) to severe (OR = 17.00, 95% CI = 15.60–24.88) health instability.

Compared to residents who did not receive hospice care and were alive 1 year following the assessment, residents who received hospice care and died within 1 year were more likely to be 95 years and older (OR = 1.31, 95% CI = 1.10–1.56), have a diagnosis of cancer (OR = 8.96, 95% CI = 8.27–9.91), and severe physical impairment (OR = 5.38, 95% CI = 2.12–13.64), and were also more likely have mild-moderate (OR = 2.03, 95% CI = 1.82–2.28) to severe (OR = 2.68, 95% CI = 2.19–3.27) pain, moderate to very high pressure ulcer risk (OR ranges from 2.18 [95% CI = 1.45–3.29] in moderate pressure ulcer risk category to 3.50 [95% CI = 2.24–4.38] in very high pressure ulcer risk category), and mild (OR = 10.40, 95% CI = 6.67–16.22) to moderate-severe (OR = 205.37, 95% CI = 132.09–319.30) health instability.

## Discussion

The assessed use rate of hospice care in LTCFs was very low (i.e., less than 3%), while one in five residents died within 3 months and one in three died within 1 year. Most deaths occurred 3 months after the assessment regardless of hospice use. Among those who did not receive hospice care, more than a quarter died within 1 year. This indicates over one in four residents in LTCFs who had potential to benefit from hospice care may not have received it. The actual use of hospice care among residents in LTCFs is critically low in Canada, which indicates an urgent and immediate need for action to improve hospice care utilization in LTCFs in Canada.

This study is one of the first to quantify and compare those who received hospice care and those who did not with proximity to death at a national level. The findings of this study indicate how the residents’ predisposing, enabling, and need characteristics influence their utilization of hospice care services, which helps to identify access disparities and other barriers to hospice use in LTCFs in Canada. The results indicate there are substantial differences in residents’ predisposing, enabling, and need characteristics among the four groups. Younger age, living in an urban LTCF, having a diagnosis of cancer, and having more severe, complex, and acute clinical needs significantly increased the likelihood of hospice use among residents in LTCFs in Canada.

The analysis of the study findings further indicates that residents who were older were more likely to die without hospice care, which reveals evidence of ageism in relation to equitable access to hospice care in LTCFs. Evidence of inequalities in access to end-of-life care, particularly between age groups, has been reported in the United States, the United Kingdom, and Australia [43–45]. Age inequalities in access to hospice care was also found in a study of cancer patients in the province of Nova Scotia, Canada [46]. It is well known that the Canadian population is ageing rapidly, thus, failure to recognize the role of ageism in relation to accessibility to hospice care may pose severe consequences. Ageism affects the way in which services at the end of life are often designed without reference to older LTCF residents’ needs [47, 48]. The tendency to give greater value to youth over old age and attribute negative characteristics to older LTCF residents may influence the older residents’ expectations and experiences of hospice care [46, 48]. Older LTCF residents’ needs should be valued during policy development with regard to improving equitable access to hospice care within LTCFs.

In our analysis, about nine in ten residents in LTCFs had a DNR order, as compared to one in three had a DNH order. Residents who received hospice care had a higher percentage of having a DNR or DNH order in place than those who did not. The casual effect between having a DNR order and receiving hospice care cannot be determined in this study. Having a DNR order in place may lead to a higher probability of referral to hospice care or vice versa. It is possible that having severe and complex health conditions may also result in more discussion of advance care planning and higher probability of having a DNR in place to be eligible for hospice referral. However, among those who received hospice care, one in ten did not have a DNR order in place, which suggests that there is still room to improve the use of advance directives among LTCF residents who receive hospice care. Among those who received hospice care, a large percentage of residents did not have a DNH in place. It remains unknow why residents who received hospice care still wanted to be hospitalized when possible and this requires further study.

The results of this study indicate that residents living in an urban LTCF were more likely to receive hospice care regardless of their one-year survivorship and were less likely to die without hospice care. Studies have demonstrated that in addition to the ability to recognize terminal stage, for LTCF residents, access to hospice may be more influenced by the facility and its location than by the residents’ treatment preferences [49, 50]. Contributors of the rural-urban difference include lack of resources and funding (e.g. lack of equipment, low salaries, and lack of specialist geriatricians) and limited access to hospice services in rural LTCFs, compared with urban LTCFs [51–53]. As hospice requires physician referral, physician shortage and high physician turnover in rural LTCFs may create barriers of hospice referral and lower the threshold for transferring residents to hospitals, especially when LTCFs and hospitals are co-located or located within a relatively short distance of each other [53, 54]. Urban areas are more likely to have a larger number of hospice providers and closer proximity of those providers to LTCFs, thus decreasing potential barriers to hospice utilization [54]. Recently, there are some studies about hospice palliative care practices in private home of rural communities in Canada [53, 55], however, it remains unclear how hospice care works in rural LTCFs [56]. The issue of LTCF-based urban-rural disparity needs further investigation to find ways to improve access to, and provision of, hospice care services in rural LTCFs.

Findings of this study indicate although residents with a diagnosis of cancer were more likely to die without hospice care, they were also more likely to receive hospice care regardless of their one-year survivorship. However, residents with a diagnosis of dementia were less likely to receive hospice care regardless of their one-year survivorship and were more likely to die without hospice care. Few seniors living with dementia in Canada receive hospice palliative care and this is more prevalent among residents in LTCFs [57]. Residents with dementia were relatively under-served by hospices. This may be partly due to difficulty in prediction of life expectancy for persons with dementia, challenges in recognition of the terminal nature of dementia, limited uptake of advance directives among persons with dementia, and poor recognition of benefits from hospice care for persons with dementia [58–62]. It is important for health care providers and policy-makers to improve recognition of dementia as a terminal disease and increase understanding of the role of hospice services for persons with dementia so these residents can have improved access to hospice care as residents with end-stage cancer.

The clinical characteristics of residents who did not receive hospice care and were alive 1 year following the assessment confirmed general expectations that this group had less severe and complex health conditions than other groups. This study indicates that residents who received hospice care and died within 1 year had the most severe and complex clinical needs among the four groups. Residents who received hospice care but were alive 1 year following the assessment exhibited more severe acute clinical needs (i.e. pain, high risk for pressure areas, and health instability) and had less chronic clinical needs (i.e. cognitive impairment) than those who did not receive hospice care but died within 1 year. More attention should be paid to residents with chronic clinical needs (i.e. cognitive impairment) when considering hospice referral.

Residents with more frequent and intense pain were more likely to receive hospice care regardless of their one-year survivorship. Due to the limitation related to the study design, the temporal sequence between time of onset of pain and time of referral to hospice care cannot be established. Therefore, it remains unclear whether pain is one of the reasons for hospice referral or residents who received hospice care were more likely to develop pain. Many studies revealed hospice care is often targeted to dying residents with higher levels of reported pain as hospice care in LTCFs can lead to better pain assessment and management for dying LTCF residents [63–66]. Hospice care providers should recognize that residents with cognitive impairment who may not be able to self-report pain [67, 68]. Data from this study indicates residents who did not receive hospice care and died within 1 year reported less pain than those who received hospice care. Part of the reason may be good control of pain from LTCF staff. Munn et al. found LTCF staff seem well positioned to control pain for residents whose deaths were expected [69]. More possible reasons may be under-detected pain among LTCF resident without hospice care, especially among those with cognitive impairment [67, 68, 70]. On the other hand, many studies indicate that hospice positively affects and improves the assessment of symptoms including pain [63–66]. Efforts from both LTCF staff and hospice providers are needed to have pain better controlled for residents who received hospice care.

Most residents triggered more than one CAP, while those who received hospice care and died within 1 year triggered more CAPs than residents in other groups. The high number of CAPs triggered among residents who received hospice care and died within 1 year reflects high levels of complex clinical needs. Among those who died within 1 year, urinary incontinence, mood, and social relationship were the top three area in which the residents had a higher than expected rate of decline, an increased potential to improve, and symptoms that could be alleviated if problems are addressed. Among those who were still alive 1 year following the assessment, activities of daily living, urinary incontinence, and mood were the top three areas in which the residents had a higher than expected rate of decline and may have benefited from alleviated symptoms if problems were addressed. The CAPs triggered at high rates among all residents, such as urinary incontinence and mood, warrant increased attention for the majority of LTCF residents. Consideration of specific triggered CAPs in this study provide evidence to support care plan related decisions based on residents’ needs.

Although residents with health instability were more likely to die with hospice care, they were also more likely to receive hospice care regardless of their one-year survivorship. Studies have found CHESS was a good predictor of mortality for hospitalized patients and persons with neurological conditions [34]. However, this study revealed hospice care was offered to a large proportion of residents who did not die within 1 year but had severe health instability. This indicates that while CHESS may be a strong predictor of mortality, it may not be a good predictor of receipt of hospice care. The predictive model developed in Xiong's study [19] was superior than CHESS alone to predict hospice care utilization, as clinical needs were not the only determinants of hospice use.

### Strengths and limitations

This study responded to a critical gap in knowledge and is the first Canadian study to use the RAI-MDS 2.0 data to examine hospice use among LTCF residents stratified by their one-year survivorship at a national level. Andersen and Newman’s behaviors model strengthened this study. This framework specified which key variables influenced hospice use and provided a more comprehensive overview of disparities in hospice care access in LTCFs in Canada. In addition, this study used a mix of admission assessments, significant change in status full assessment, annual full assessments, and quarterly assessments. Thus, the study population covered all stage of residents living in LTCFs. Moreover, this study linked the DAD to the CCRS, which allowed the tracking of an extra 5.2% residents who were discharged from LTCFs and did not have death records in LTCFs.

Despite important strengths, this study has several limitations. First, this study showed one-year survivorship of hospice use among residents living in LTCFs in Canada at the time of their last assessments in 2015. The hospice use rate among LTCF residents at death cannot be identified, as some residents who did not receive hospice care at the time of last assessment in 2015 may have received hospice care after the assessment. Second, this study revealed substantial differences in hospice use across geographical areas. However, the differences among all provinces in Canada cannot be explicitly examined, as the CCRS database does not contain full coverage of LTCFs in all provinces of Canada although the CCRS database is the most comprehensive database available to describe the LTCF population in Canada. Manitoba, New Brunswick, and Nova Scotia are partly covered and Quebec, Prince Edward Island, the Northwest Territories, and Nunavut are not covered in the CCRS database. Third, although the study revealed numerous associations between resident characteristics and hospice use, causality could not be determined due to study design. Longitudinal study of differences of resident characteristics with an increased number of time points is recommended to better understand the association between resident characteristics and hospice use. Fourth, complete case analyses were used in the predictive modeling. To reduce potential for biased and inefficient estimates, variables with a large proportion of missing values, such as DNR order (5.8%), marital status (12.7%) and education (36.7%) were removed from predictive modeling.

## Conclusion

The actual use of hospice care among LTCF residents is very poor in Canada. Residents who received hospice care and died within 1 year of assessment exhibited more severe and complex clinical needs than those who did not receive hospice care and those who were alive 1 year following the assessment. Residents who received hospice care and were alive 1 year following the assessment exhibited more severe acute clinical needs (i.e. pain, high pressure risk, and health instability) and had less chronic clinical needs (i.e. cognitive impairment, depression, and low social engagement) than those who did not receive hospice care but died within 1 year. This study indicates several possible barriers to hospice use in the LTCF population including ageism, rurality, and a diagnosis of dementia. As Andersen and Newman’s behavior model indicates, all these factors come together to explain and inform health care utilization. Immediate action is needed to address inequality in care at the end of life for the LTCF population and provide improved access to high quality hospice care in LTCFs in Canada.

## Data Availability

The datasets generated and/or analysed during the current study were obtained from the Canadian Institute for Health Information. The authors do not have the permission to share the datasets.

## Abbreviations

CCRS: Canadian Continuing Reporting System
DAD: Discharge Abstract Database
LTCF: Long-term care facilities
RAI-MDS 2.0: Resident Assessment Instrument Minimum Data Set Version 2.0

## References

[1] Freeman S, Bishop K, Spirgiene L, Koopmans E, Bothelo FC, Fyfe T (2017). Factors affecting residents transition from long-term care facilities to the community: a scoping review. BMC Health Serv Res.

[2] Carstairs S (2010). Raising the bar: a roadmap for the future of palliative care in Canada.

[3] Hirdes JP, Mitchell L, Maxwell CJ, White N (2011). Beyond the ‘iron lungs of gerontology’: using evidence to shape the future of nursing homes in Canada. Can J Aging.

[4] Jayaraman J, Joseph KS (2013). Determinants of place of death: a population-based retrospective cohort study. BMC Palliat Care.

[5] McGregor MJ, Ronald LA (2011). Residential long-term care for Canadian seniors: nonprofit, for-profit or does it matter?.

[6] Health Canada (2004). Long-term facilities-based care.

[7] Centre for Health Services and Policy Research (2015). Long-term care - evidence and perspectives on funding healthcare in Canada.

[8] Canadian Hospice Palliative Care Association (2016). What is palliative care?.

[9] Canadian Virtual Hospice (2019). How is eligibility for palliative care decided? What procedure is followed?.

[10] Canadian Institute for Health Information (CIHI) (2015). Resident Assessment Instrument (RAI) MDS 2.0 user’s manual, Canadian version.

[11] Langille J (2013). Rights of passage: integrating palliative care. Can Nurse.

[12] Hawley PH (2014). The bow tie model of 21st century palliative care. J Pain Symptom Manag.

[13] Health Canada (2016). Palliative care.

[14] Infeld DL, Crum GE, Koshuta MA (1990). Characteristics of patients in a long-term care hospice setting. Hosp J.

[15] Petrisek AC, Mor V (1999). Hospice in nursing homes: a facility-level analysis of the distribution of hospice beneficiaries. Gerontologist..

[16] Castle NG (1999). Hospice and nursing homes. J Health Soc Policy.

[17] Quality Palliative Care in Long-Term Care Alliance (2011). Long-term care homes: Hospices of the future.

[18] Brink P, Kelley ML (2015). Death in long-term care: a brief report examining factors associated with death within 31 days of assessment. Palliat Care.

[19] Xiong B. Factors affecting hospice care use among long-term care facility residents in Canada. 2019. 10.24124/2019/58970. Accessed 11 Nov 2019.

[20] Ersek M, Carpenter JG (2013). Geriatric palliative care in long-term care settings with a focus on nursing homes. J Palliat Med.

[21] Andersen RM, Newman JF (1973). Societal and individual determinants of medical care utilization in the United States. Milbank Mem Fund Q.

[22] Li YN, Nong DX, Wei B, Feng QM, Luo HY (2016). The impact of predisposing, enabling, and need factors in utilization of health services among rural residents in Guangxi, China. BMC Health Serv Res.

[23] Lo KM, Fulda KG (2008). Impact of predisposing, enabling, and need factors in accessing preventive medical care among U.S. children: Results of the national survey of children’s health. Osteopath Med Prim Care.

[24] CIHI (2017). Continuing care reporting system data submission user manual.

[25] CIHI (2018). Continuing care metadata.

[26] CIHI (2015). Resident Assessment Instrument (RAI) MDS 2.0 user’s manual, Canadian version.

[27] CIHI (2018). Discharge abstract database metadata.

[28] Statistics Canada (2017). Population Centre and rural area classification 2016.

[29] Public Health Ontario (2013). Summary measures of socioeconomic inequalities in health.

[30] Morris JN, Fries BE, Mehr DR, Hawes C, Phillips C, Mor V, Lipsitz LA (1994). MDS cognitive performance scale. J Gerontol.

[31] Burrows AB, Morris JN, Simon SE, Hirdes JP, Phillips CD (2000). Development of an MDS-based depression rating scale for use in nursing homes. Age Ageing.

[32] Mor V, Branco K, Fleishman J, Hawes C, Phillips C, Morris J, Fries B (1995). The structure of social engagement among nursing home residents. J Gerontol B Psychol Sci Soc Sci.

[33] Landi F, Tua E, Onder G, Carrara B, Sgadari A, Rinaldi C (2000). Minimum data set for home care: a valid instrument to assess frail older people living in the community. Med Care.

[34] Hirdes JP, Frijters DH, Teare GF (2003). The MDS-CHESS scale: a new measure to predict mortality in institutionalized older people. J Am Geriatr Soc.

[35] Perlman CM, Hirdes JP (2008). The aggressive behavior scale: a new scale to measure aggression based on the minimum data set. J Am Geriatr Soc.

[36] Fries BE, Simon SE, Morris JN, Flodstrom C, Bookstein FL (2001). Pain in the U.S. nursing homes: validating a pain scale for the minimum data set. Gerontologist..

[37] Poss JW, Murphy KM, Woodbury MG, Orsted HL, Stevenson K, Williams GD (2010). Development of the interRAI pressure ulcer risk scale (PURS) for use in long-term care and home care settings. BMC Geriatr.

[38] interRAI (2017). Understanding LTCF Clinical Assessment Protocols (CAPs).

[39] Canadian Institute for Health Information (CIHI) (2008). interRAI Clinical Assessment Protocols (CAPs)—for use with interRAI’s community and long-term care assessment instruments.

[40] Freeman S, Hirdes JP, Stolee P, Garcia J, Smith TR, Steel K, Morris JN (2014). Care planning needs of palliative home care clients: development of the interRAI palliative care assessment clinical assessment protocols (CAPs). BMC Palliat Care.

[41] Tabachnick BG, Fidell LS (2012). Using multivariate statistics.

[42] Hair JF, Black WC, Babin BJ, Anderson RE (2010). Multivariate data analysis.

[43] Addington-Hall J (2000). Which terminally ill cancer patients in the United Kingdom receive care from community specialist palliative care nurses?. J Adv Nurs.

[44] Burt J, Raine R (2006). The effect of age on referral to and use of specialist palliative care services in adult cancer patients: a systematic review. Age Ageing.

[45] Rosenwax LK, McNamara BA (2006). Who receives specialist palliative care in Western Australia–and who misses out. J Palliat Med.

[46] Burge FI, Lawson BJ, Johnston GM, Grunfeld E (2008). A population-based study of age inequalities in access to palliative care among cancer patients. Med Care.

[47] Gardiner C, Cobb M, Gott M, Ingleton C (2011). Barriers to providing palliative care for older people in acute hospitals. Age Ageing.

[48] Gott M, Ibrahim AM, Binstock RH, Gott M, Ingleton C (2011). The disadvantaged dying: ageing, ageism, and palliative care provision for older people in the UK. Living with ageing and dying.

[49] Zerzan J, Stearns S, Hanson L (2000). Access to palliative care and hospice in nursing homes. JAMA..

[50] Welch LC, Miller SC, Martin EW, Nanda A (2008). Referral and timing of referral to hospice care in nursing homes: the significant role of staff members. Gerontologist..

[51] Virnig BA, Hartman L, Moscovice I, Carlin B (2006). Access to home-based hospice care for rural populations: identification of areas lacking service. J Palliat Med.

[52] Kelley ML, Sletmoen W, Williams AM, Nadin S, Puiras T, Kulig JC, Williams AM (2012). Integrating policy, research, and community development: a case study of developing rural palliative care. Health in rural Canada.

[53] Kaasalainen S, Brazil K, Williams A, Wilson D, Willison K, Marshall D, Taniguchi A (2012). Barriers and enablers to providing palliative care in rural communities: a nursing perspective. J Rural Community Dev.

[54] Temkin-Greener H, Zheng NT, Mukamel DB (2012). Rural-urban differences in end-of-life nursing home care: facility and environmental factors. Gerontologist..

[55] Whitfield KY (2018). A case study exploring the implications of one Alberta rural community’s experience with planning their own hospice care. J Rural Community Dev.

[56] DeMiglio L, Dykeman S, Williams A, Kelley ML (2012). Evolution of palliative care in Ontario: the impact of geography, funding, and advocacy. J Rural Community Dev.

[57] CIHI (2019). Seniors living with dementia in Canada facing gap in palliative care.

[58] Erel M, Marcus EL, Dekeyser-Ganz F (2017). Barriers to palliative care for advanced dementia: a scoping review. Ann Palliat Med.

[59] Mitchell S, Kiely D, Hamel M (2004). Dying with advanced dementia in the nursing home. Arch Intern Med.

[60] Mitchell SL, Miller SC, Teno JM, Kiely DK, Davis RB, Shaffer ML (2010). Prediction of 6-month survival of nursing home residents with advanced dementia using ADEPT vs. hospice eligibility guidelines. JAMA..

[61] Sachs GA, Shega JW, Cox-Hayley D (2004). Barriers to excellent end-of-life care for patients with dementia. J Gen Intern Med.

[62] Kiely DK, Givens JL, Shaffer ML, Teno JM, Mitchell SL (2010). Hospice use and outcomes in nursing home residents with advanced dementia. J Am Geriatr Soc.

[63] Miller SC, Mor V, Teno J (2003). Hospice enrollment and pain assessment and management in nursing homes. J Pain Symptom Manag.

[64] Miller SC, Mor V, Wu N, Gozalo P, Lapane K (2003). Does receipt of hospice care in nursing homes improve the management of pain at the end of life?. J Am Geriatr Soc.

[65] Baer WM, Hanson LC (2000). Families’ perception of the added value of hospice in the nursing home. J Am Geriatr Soc.

[66] Wu N, Miller SC, Lapane K, Gozalo P (2003). The problem of assessment bias when measuring the hospice effect on nursing home residents’ pain. J Pain Symptom Manag.

[67] Herr K, Coyne PJ, McCaffery M, Manworren, Merkel S (2011). Pain assessment in the patient unable to self-report: position statement with clinical practice recommendations. Pain Manag Nurs.

[68] Miu DKY, Chan KC (2014). Under-detection of pain in elderly nursing home residents with moderate to severe dementia. J Clin Gerontol Geriatr.

[69] Munn JC, Hanson LC, Zimmerman S, Sloane PD, Mitchell CM (2006). Is hospice associated with improved end-of-life care in nursing homes and assisted living facilities?. J Am Geriatr Soc.

[70] Marx TL (2005). Partnering with hospice to improve pain management in the nursing home setting. J Am Osteopath Assoc.

